# Generation of infrared photon pairs by spontaneous four-wave mixing in a CS_2_-filled microstructured optical fiber

**DOI:** 10.1038/s41598-024-51482-0

**Published:** 2024-01-10

**Authors:** Mina Afsharnia, Saher Junaid, Sina Saravi, Mario Chemnitz, Katrin Wondraczek, Thomas Pertsch, Markus A. Schmidt, Frank Setzpfandt

**Affiliations:** 1 Institute of Applied Physics, Abbe Center of Photonics, Friedrich Schiller University Jena, Albert-Einstein-Straße 15, 07745 Jena, Germany; 2 Fraunhofer Institute for Applied Optics and Precision Engineering IOF, Albert-Einstein-Str. 7, 07745 Jena, Germany; 3 Leibniz Institute of Photonic Technology, Albert-Einstein-Street 9, 07745 Jena, Germany; 4 Abbe Center of Photonics and Faculty of Physics, Friedrich-Schiller-University Jena, Max-Wien-Platz 1, 07743 Jena, Germany; 5 Otto Schott Institute of Materials Research, Friedrich Schiller University Jena, Fraunhoferstr. 6, 07743 Jena, Germany

**Keywords:** Nonlinear optics, Quantum optics

## Abstract

We experimentally demonstrate frequency non-degenerate photon-pair generation via spontaneous four-wave mixing from a novel CS_2_-filled microstructured optical fiber. CS_2_ has high nonlinearity, narrow Raman lines, a broad transmission spectrum, and also has a large index contrast with the microstructured silica fiber. We can achieve phase matching over a large spectral range by tuning the pump wavelength, allowing the generation of idler photons in the infrared region, which is suitable for applications in quantum spectroscopy. Moreover, we demonstrate a coincidence-to-accidental ratio of larger than 90 and a pair generation efficiency of about $10^{-2}$ per pump pulse, which shows the viability of this fiber-based platform as a photon-pair source for quantum technology applications.

## Introduction

Several quantum optics applications, such as quantum imaging and spectroscopy, rely on quantum-correlated photon pairs with non-degenerate wavelengths. These applications include detecting one photon, whereas its correlated partner photon probes the test object at a different wavelength^1–4^. Thus, a photon-pair source providing pairs with one photon in the visible and one in the infrared is a powerful tool to perform measurements in the infrared spectral range without using an infrared detector, as the object information can be obtained using highly efficient and well-developed visible range detectors. Accessing the mid-infrared (MIR) spectral range is important as it covers the unique fingerprints of many organic and inorganic materials. Therefore, for many applications such as quantum imaging^1^, microscopy^5^, spectroscopy^4,6,7^, and optical coherence tomography^3^, developing high-quality correlated photon pair sources to generate highly non-degenerate and widely tunable correlated photon pairs with idler photons in the MIR spectral range is essential. Optical fiber sources based on spontaneous four-wave mixing (SFWM) are one of the brightest photon-pair sources, as fibers can be drawn in long lengths to provide meters of light-matter interaction length^8–10^. They are also ideal for integration with optical-fiber networks because of the comparable mode shapes of the sources and networks. As any long-distance quantum network requires fibers, a fiber-based source that could be directly spliced to the existing fiber network is desirable^8,11^. In this regard, silica-core fibers have attracted considerable attention due to the simplicity of their implementation^12–16^. Photon-pair generation using photonic crystal fibers (PCF)^17^, dispersion-shifted fibers^18,19^, step-index multimode optical fibers (MMF)^20^, graded-index MMF^21^, and birefringent fibers^22^ has been reported as well. Among these optical fiber alternatives, PCFs and microstructured fibers exhibit unique characteristics that make them an attractive medium for photon-pair source implementations^9,23–26^. They exhibit engineered dispersion properties enabling spectrally or temporally tailored two-photon state generation, and high effective nonlinear coefficients permitting large emission rates. Nevertheless, silica-core fibers have low nonlinearity and limited transmission bandwidth. Besides, studies about generating correlated photon pairs via spontaneous four-wave mixing (SFWM) in silica core and microstructured fibers indicated that these sources were limited regarding quantum purity due to spontaneous Raman scattering (SpRS)^11,27–29^. Because of the very broad Raman spectrum of silica^30^ no matter which wavelengths are emitted by the correlated photons, some uncorrelated Raman photons are also emitted at these wavelengths in the silica core fiber and degrade the quality of the source. One approach that has been investigated to reduce the Raman noise is cooling down the device^18,29^. However, this method cannot completely suppress the Raman noise and adds a layer of experimental difficulty. Another approach compatible with room-temperature operation is generating photon pairs with a large spectral gap from the pump. This approach has been mostly investigated in specially designed microstructured fibers^31^. Although Raman noise becomes less detrimental, multiphonon Raman scattering can still be troublesome^23,32^. An ideal solution is to change the propagation medium to a material exhibiting a narrow-line Raman spectrum. Microstructured optical fibers (MOF) allow to guide light in a gas or a liquid with a negligible optical overlap with the glass. Several demonstrations have shown the ability to explore Raman-free nonlinear optics^33–37^.

A promising approach to overcome the limitations of silica-core fibers is generating photon-pairs in liquid-core optical fibers (Li-COF)^38^. M. Barbier et al experimentally demonstrated the generation of correlated photon pairs in a deuterated acetone-core photonic crystal fiber. The liquid-core optical fibers have also been used for other nonlinear experiments^39,40^ such as supercontinuum generation^41–46^ and third-harmonic generation^47^. Among different liquids, carbon disulfide (CS_2_) attracts most attention^48,49^, as CS_2_ offers a significantly high nonlinearity, allowing for shorter-length fiber sources. It also has narrow Raman lines, wider transmission windows, and a tunable dispersion through mixing with other liquids^50,51^. Wider transmission windows, particularly towards mid-infrared wavelengths, enable producing photons with longer wavelengths, useful for quantum spectroscopy applications^4,6,7^. Moreover, by tuning the working temperature^52^, choosing the proper fiber geometry and the linear refractive index of the liquid through mixing different liquids, it becomes possible to engineer the dispersion relation^52,53^ and generate photon pairs outside the narrow Raman lines of the liquid^36,54^. As a result, the Raman photons generated in the core of the fiber can be filtered out as their wavelengths are well separated from those of the emitted correlated photons.

**Figure 1:**
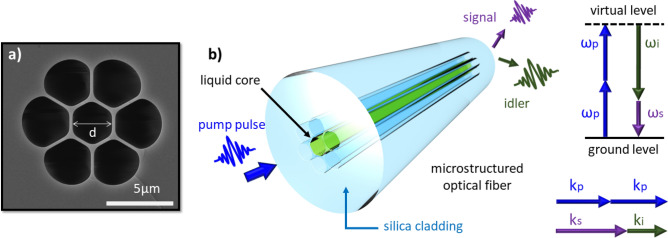
The scanning electron microscope (SEM) image and schematic of the microstructured optical fiber. (**a**) The scanning electron microscope (SEM) image of the fiber with the core diameter of d $\approx$ 2.8 $\upmu$m. The silica web surrounding the core has a thickness of approximately 0.25 $\upmu$m. (**b**) Schematic of the liquid core microstructured optical fiber and the SFWM process, in which two pump photons are annihilated in the medium and then produce a signal and idler photon.

According to these previous works, generating photons outside the Raman lines from a liquid-core optical fiber is possible^36^. Here, we will apply this concept to a novel CS_2_-filled microstructured fiber for photon-pair generation with highly non-degenerate frequencies, which allows producing idler photons in the infrared region, suitable for applications in quantum spectroscopy. The proposed fiber consists of a micrometer-sized hollow core that can be selectively filled with liquid CS_2_; the core is surrounded by air holes. The scanning electron microscope (SEM) image of an example fiber is shown in Fig. 1a, while Fig. 1b shows a sketch of the proposed fiber photon pair source. This structure offers strong transverse spatial mode confinement in the small liquid-filled core, caused by high dielectric contrast. Moreover, since the refractive index of silica is lower than the refractive index of CS_2_, no guided modes are formed in the glass encapsulating the liquid.

In this paper, we experimentally demonstrate the generation of frequency non-degenerate correlated photon pairs in a novel CS_2_-core microstructured optical fiber. Our results contribute to developing high-quality photon-pair sources and the developed source offers a potential application as a compact and efficient source for quantum spectroscopy. This paper is organized as follows: in “Spontaneous four-wave mixing theory” section, we briefly introduce and review previously developed concepts for the theory of SFWM in birefringent fibers; in “Experimental implementation” section, we present details of the experimental implementation used to generate and detect the correlated photon pairs, and in “Results and discussion” section, we discuss our experimental results. Finally, in “Conclusion” section, we summarize and conclude our results.

## Spontaneous four-wave mixing theory

Spontaneous four-wave mixing (SFWM) is a third-order nonlinear process in which two photons from the pump field with frequency $\omega$_p_ are annihilated to create a pair of photons. Throughout this paper, we assume that the two pump fields have the same spectrum. One of the generated photons is called the signal photon with frequency $\omega$_s_, and the other one is the idler photon with frequency $\omega$_i_. This phase-sensitive process is most efficient when the energy and momentum conservation conditions are satisfied, i.e.

1 $$\begin{aligned}{} & {} 2\omega _p = \omega _s + \omega _i, \end{aligned}$$

2 $$\begin{aligned}{} & {} \Delta K(\omega _s,\omega _i) = 2k(\omega _p) - k(\omega _s) - k(\omega _i) = 0, \end{aligned}$$

where $k(\omega )$ is the wave vector of each corresponding mode and $\Delta K$ is the wave-vector mismatch. We also assume low pump powers such that self- and cross-phase modulation are negligible, thus the phase-matching (PM) condition does not depend on power.

**Figure 2:**
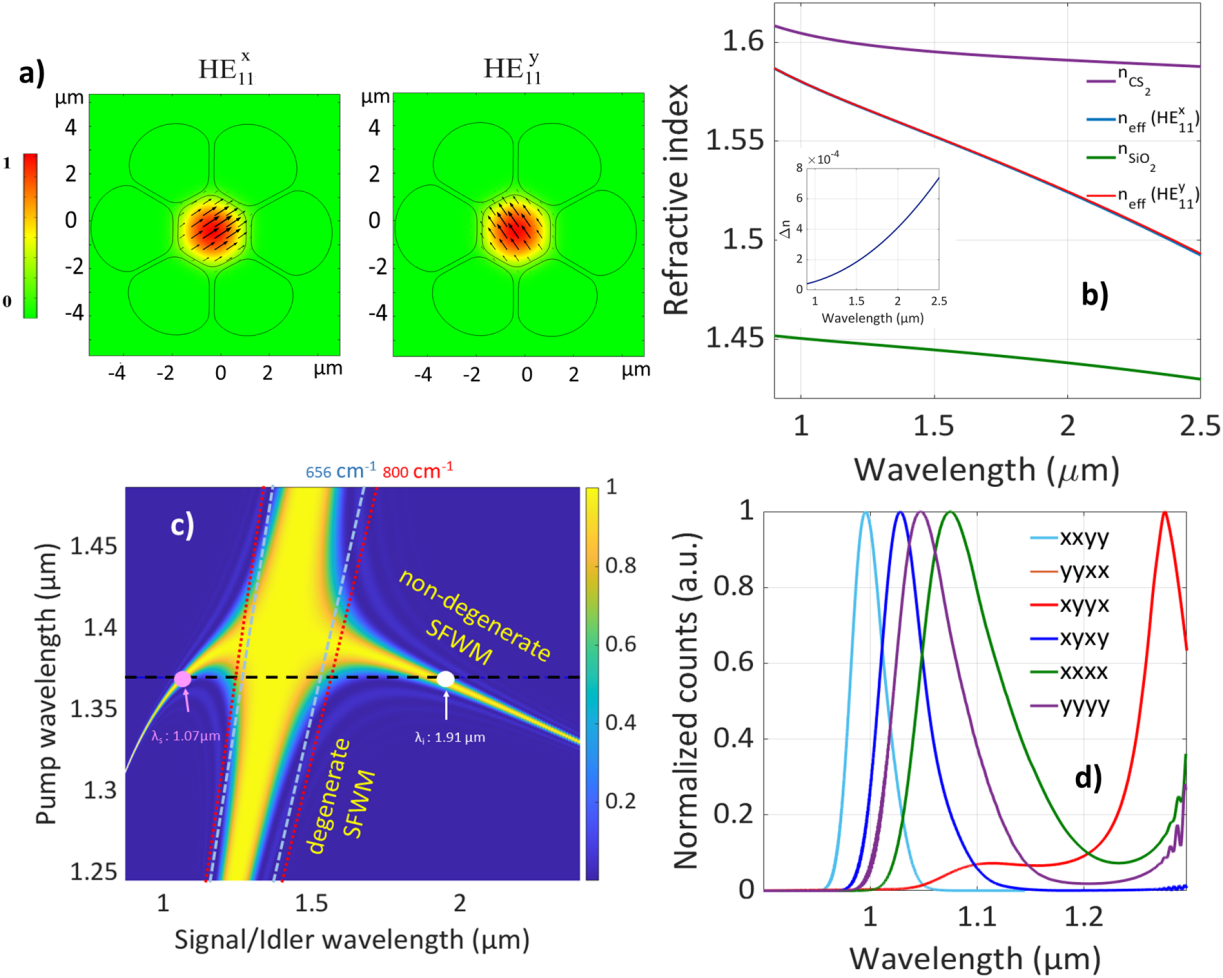
Simulation result of the CS_2_-filled microstructured optical fiber. (**a**) Profiles of the x- and y-polarized fundamental modes. For the pump wavelength at 1.37 $\upmu$m, the effective mode area is approximately 4.5 $\upmu$m^2^. (**b**) The refractive index of CS_2_, silica, and effective refractive index for the x- and y-polarized fundamental modes. Inset shows the index difference between these two modes. (**c**) ${{\,\textrm{sinc}\,}}^2 [ \Delta K (\omega _s,\omega _i) {L}/{2}]$ for x-polarized fundamental signal, idler, and pump modes, and the Horizontal black dashed line represents a CW pump at 1.37 $\upmu$m. Blue and red dashed lines correspond to Raman lines of CS_2_ for different pump wavelengths. They correspond to the Stokes ($\lambda > \lambda _p$) and anti-Stokes ($\lambda < \lambda _p$) lines. (**d**) Calculated signal spectra for the six SFWM processes that incorporate the measured pump pulse spectra.

In this work, we study the SFWM process in a CS_2_-filled microstructured optical fiber. We modeled this fiber using the finite element method based on geometrical parameters extracted from the SEM image shown in Fig. 1a. From that, we found two orthogonally polarized fundamental modes. Although this fiber is capable of supporting higher-order modes, particularly at the shortest working wavelength (signal wavelength), our study exclusively concentrates on utilizing the fundamental modes for pumping and pair generation, due to their higher in- and out-coupling efficiencies. This focus on the fundamental mode enables us to achieve elevated incoupling efficiency for the pump and enhanced outcoupling efficiency for both the signal and idler. The polarization direction of the fundamental modes is shown in their mode profiles in Fig. 2a. We call the polarization direction shown in the left mode profile x and the other one y. Therefore, this fiber supports two orthogonally polarized fundamental modes: the x-polarized fundamental mode ( HE$_{11}^x$ ) and the y-polarized fundamental mode (HE$_{11}^y$).

The numerical modeling of the effective refractive index (n_eff_) and mode profile for this fiber was carried out for both fundamental modes. The wavelength-dependent refractive indices of silica and CS_2_ are included through the Sellmeier equations^55–57^. Figure 2b shows the refractive index of CS_2_, silica, and the calculated effective refractive indices for the x-polarized fundamental mode and the y-polarized fundamental mode. The n_eff_ for these two fundamental modes are very close to each other. For instance, at $\lambda _p=1.37$ $\upmu$m, the index difference between these two modes is $1.5 \times 10^{-4}$. For smaller wavelengths, the mode is more confined to CS_2_ and the modal characteristics are mainly determined by the CS_2_-filled core, so n_eff_ is closer to the refractive index of CS_2_ because of the high index contrast and the resulting strong confinement. When the wavelength is longer, the mode expands more to the silica-air area. In such a case, the mode is influenced by both the CS_2_-filled core and the silica. This leads to a lower n_eff_. For longer wavelengths, it is essential to note that an increased fraction of the field is overlapping with silica, which is an important factor for loss consideration at longer wavelengths. In this study, the generated wavelengths, particularly the longer one (idler), remain below 2 $\upmu$m, where silica losses are negligible. Nonetheless, for wavelengths exceeding 2.5 $\upmu$m, silica absorption becomes significant, resulting in increased propagation losses.

The efficiency of the SFWM process is proportional to ${{\,\textrm{sinc}\,}}^2 [ \Delta K (\omega _s,\omega _i) {L}/{2}]$, where *L* is the length of SFWM medium and $\Delta K$ is the phase mismatch^58^. Figure 2c shows ${{\,\textrm{sinc}\,}}^2 [ \Delta K (\omega _s,\omega _i) {L}/{2}]$ as a function of pump wavelengths for a fiber length of L = 15 cm. We assume that the pump, signal, and idler are all in the x-polarized fundamental mode. For efficient SFWM, the phase mismatch must be zero ($\Delta K = 0$), which maximizes the ${{\,\textrm{sinc}\,}}^2 [ \Delta K (\omega _s,\omega _i) {L}/{2}]$ function. This curve comprises two parts, the diagonal line corresponding to degenerate SFWM and the parabolic curve corresponding to non-degenerate SFWM. In this paper, degenerate and non-degenerate terms are referring to the generated photon’s wavelength. The crossing point corresponds to the zero dispersion wavelength (ZDW) of the x-polarized fundamental mode ( HE$_{11}^x$ ). In the non-degenerate case, generated photon pairs are well separated from both the residual pump photons and the spontaneous Raman scattering (SpRS). Raman lines of CS_2_ (656 cm$^{-1}$ and 800 cm$^{-1}$)^59^ for different pump wavelengths are shown in Fig. 2c. Furthermore, guiding light in the CS_2_ core contributes to a reduction of the Raman noise in photon-pair generation. For instance, in this fiber at $\lambda _p=1.37$ $\upmu$m, only 2% of the energy is interacting with silica, showing a small optical overlap with silica compared to an all-silica fiber with similar dimensions. This fiber also has other advantageous features. The used CS_2_ core features a very high nonlinearity, resulting in much higher effective nonlinear coefficients than for commonly employed fibers. This is further improved by the strong localization in our fiber design, such that the effective nonlinearity^30,60^ for our fiber parameters is $\gamma \approx$ 1 W^-1^ m^-1^, several orders of magnitude higher than in conventional silica fibers and even higher than in CS_2_-filled fibers used for supercontinuum generation^41^. Moreover, we can achieve phase-matching over a large spectral range by tuning the pump wavelength. This allows for the generation of idler photons in the infrared region, suitable for quantum spectroscopy and sensing applications.

Due to the presence of the two orthogonally polarized fundamental modes in the structure, the SFWM process can occur in various polarization combinations amongst the four fields involved. It can be shown that the SFWM interaction Hamiltonian^61^ comprises six different processes related to different polarization combinations of the participating fields, each with a corresponding coefficient^62^. Therefore, the two-photon state is the coherent superposition of the six different polarization combinations. Table 1 shows the list of the SFWM processes related to different polarization combinations of the participating electric fields.

**Table 1:** List of the SFWM processes for different electric field polarization combinations.

$\textbf{Process}$	$\textbf{p}$_1_	$\textbf{p}$_2_	$\textbf{s}$ ($\lambda$_p_ > $\lambda$_s_)	$\textbf{i}$ ($\lambda$_i_ > $\lambda$_p_)
1	x	x	x	x
2	y	y	y	y
3	x	y	x	y
4	x	y	y	x
5	x	x	y	y
6	y	y	y	y

List of the SFWM processes related to different polarization combinations of the participating electric fields^62^. p_1_, p_2_, s and i correspond to pump_1_, pump_2_, signal and idler.

Here, the SFWM pump is defined by its optical frequency and polarization. On one hand, it is evident from Table 1, that the pump polarization controls which of the six processes may occur. On the other hand, it is clear from Fig. 2c that by spectrally tuning the pump frequency, one can influence the resulting signal and idler frequencies according to the phase-matching contours. This dependence of the emission characteristics on the pump polarization and frequency may be used as a means to control the two-photon state. In Fig. 2d, we show the calculated signal spectrum for all six processes. For this calculation, we used the measured laser pump pulse with the central wavelength at $\lambda _p=1.37$ $\upmu$m. Details on the calculation can be found in references^52,63^. For the wavelength range shown in Fig. 2d, phase-matching is fulfilled for all processes except yyxx. Note that at certain pump frequencies, the phase-matching contour arches in such a manner that leads to a dual peak for each of the signal and idler, as indeed happens for the xyyx process at $\lambda _p=1.37$ $\upmu$m. In this case, the two-photon state is composed of two distinct spectral zones, resulting from two separate solutions to $\Delta K = 0$. Thus, this process produces a double spectral peak within the signal/idler spectral windows used in Fig. 2d. As the six processes correspond to different combinations of polarizations, and the dispersion relations depend on polarization, the frequencies at which phase-matching is attained for these six processes tend to be different. However, due to the bandwidth of the pump pulse, the signal spectra of these six SFWM processes overlap each other and we cannot distinguish them by spectral filtering, so we use polarization to distinguish a specific process. In this work, we characterize our fiber-based photon pair source for only one polarization combination which is process xxxx. In this process, all the components are considered to be in the x-polarized fundamental mode. We focus on this process because the co-polarized FWM is roughly nine times more efficient than the orthogonally polarized one^64^.

## Experimental implementation

### Selective filling of microstructured fiber with carbon disulfide liquid

We have used a specially designed microstructured fiber with seven air holes, where the central one (core) is filled with CS_2_. Different techniques have been employed for the selective filling of microstructured fibers^53,65–67^. Here, the selective filling of liquid into the central hole of the microstructured fiber is achieved using pressure-assisted collapse (PAC). This procedure for selective filling is exploited for microstructured fibers when core and cladding air holes have equal size and a splicer-arc cannot be used to collapse cladding holes, as presented in reference^53^. Therefore, PAC is used by generating a pressure difference between core and cladding air holes, which helps in collapsing the outer air holes with low air pressure using a splicer-arc, while keeping the central hole with high-pressure open. Note that the length of the section where the fiber cross-section is actually affected (in the order of 150 $\upmu$m) is short and has negligible effect on the photon-pair generation dynamics that occur over tens of centimeters.

**Figure 3:**
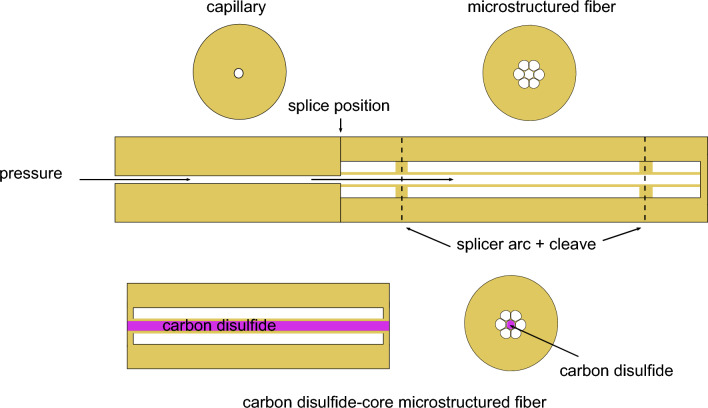
Filling procedure of the CS_**2**_-filled microstructured optical fiber. Sketch of the procedure of selective filling of microstructured fiber with carbon disulfide liquid.

Figure 3 shows the schematic of the fiber preparation process where the yellow color represents silica glass, white is air, and pink is used to illustrate CS_2_. The selective filling is done following these steps:

*Step 1*: A silica capillary with an inner diameter of 1.6 $\upmu$m is spliced to the core of the microstructured fiber while the hole of the capillary is precisely aligned with the central hole of the microstructured fiber. The other end of the microstructured fiber is completely collapsed.
*Step 2*: Gas (Nitrogen) pressure (approx. 5 bar) is applied in the central hole of the microstructured fiber via the spliced capillary, which builds high pressure solely in the central hole of the microstructured fiber.
*Step 3*: A splicer-arc is applied to the microstructured fiber, which leads to collapsing of all outer holes of the fiber at the splice position. This selective collapse is done at two positions depending on the desired length of the fiber sample. The process of cleaving, which involves precise cutting, is performed at the location of the collapse.
*Step 4*: The prepared fiber is mounted in two custom-built optofluidic mounts (OFM, i.e. small tanks with a capacity of approximately 1 $\upmu$l per mount, made of aluminum sealed with optical windows (1 mm thick sapphire window) and inlet, outlet and fiber ports)^68,69^. Note that we do not observe any effect of the birefringence of the sapphire windows in our experiments. In each OFM the fiber body is sealed by a sleeve-ferrule connection inside the fiber port. Upon subsequent filling of the OFMs, the liquid fills the central hole of the microstructured fiber using capillary action.

### Experimental setup

**Figure 4:**
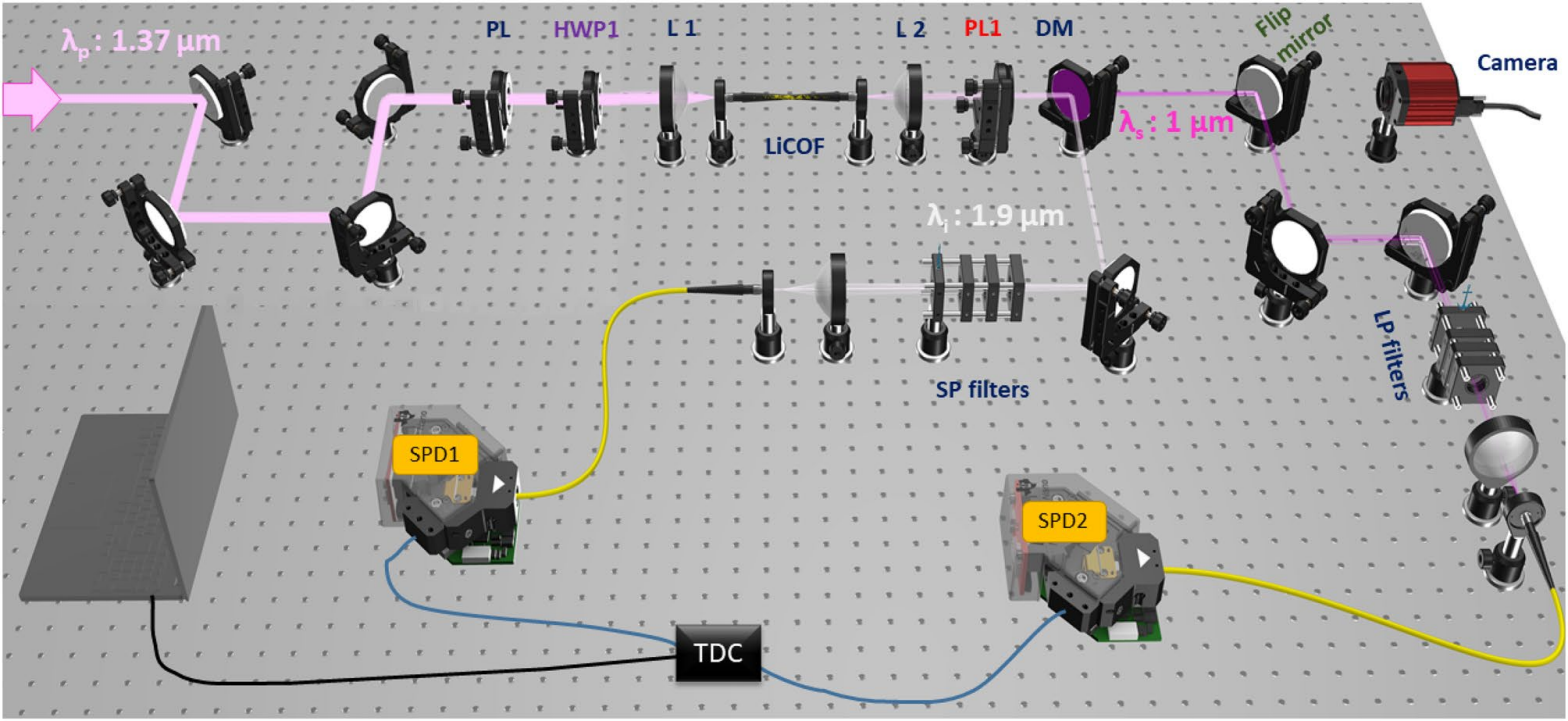
Schematic of the experimental setup. Experimental setup for characterizing photon-pairs generated in a CS_2_-filled microstructured optical fiber through SFWM. *HWP* Half-wave plate, *QWP* Quarter-wave plate, *L* Aspherical lens, *PL* Polarizer, *DM* Dichroic mirror, *LiCOF* Liquid-core optical fiber, *SP filter* Short-pass filter, *LP filter* Long-pass filter, *SPD* Single photon detector, *TDC* Time-to-digital converter.

Our experimental setup is depicted in Fig. 4. The nonlinear waveguide is a CS_2_-filled microstructured optical fiber, with a length of 15 cm. Pump pulses are delivered from a tunable femtosecond mode-locked Ti:Sapphire (Ti:Sa) pumped OPO laser with a repetition rate of 80 MHz and pulse duration of 225 fs. We use an excitation pump with a Gaussian beam profile which is well-matched to the fundamental mode of the fiber. Therefore, with a fine adjustment, one can stably inject the pump pulses in the fundamental mode by maximizing the intensity in the center of the core (as was checked in our experiments by regularly imaging the output face of the fiber on a CCD camera). We measure approximately a 10% coupling efficiency through our fiber for the pump beam. It is important to note that, in the context of this study, the term ’pump power’ consistently refers to the incident pump power. For pump wavelength, we chose $\lambda _p$ = 1370 nm, which leads to the generation of signal and idler photons at 1 $\upmu$m and 1.9 $\upmu$m, respectively. A combination of a half-wave plate (HWP) and a polarizer is used to control the input pump power and polarization. The pump polarization angle can be adjusted with the help of a half-wave plate (HWP1) so that it matches the x- or y-axis or any other direction. Thus, when the pump field is linearly polarized parallel to the x-axis, emitted photons must be either both x-polarized (this corresponds to process xxxx) or both y-polarized (process xxyy), see Table 1. Considering our emphasis on the xxxx process, another polarizer is placed after the liquid-core fiber (PL1). This polarizer is employed to separate the generated signal and idler photons with the x-polarization.

Fiber incoupling and outcoupling of the signal and idler photons are accomplished with aspheric lenses (L1) and (L2). As depicted in Fig. 4, photons emerging from L2 are split into two paths using a dichroic mirror (DM), which reflects wavelengths $\lambda<$ 1500 nm and transmits wavelengths $\lambda>$ 1500 nm. This dichroic mirror is used to separate the pump photons and spontaneously generated signal photons from idler photons.

By adequately choosing the pump wavelength for the SFWM process, we avoid the two Raman lines of CS_2_ (656 cm$^{-1}$ and 800 cm$^{-1}$)^59^. Raman lines of CS_2_ for different pump wavelengths are shown in Fig. 2c. We used shortpass filters in the signal arm and longpass filters in the idler arm to suppress pump photons. The presence of five long-pass filters with the cut-on wavelength of 1650 nm in the idler arm ensures that only photons above 1650 nm wavelength can reach the idler detector. At the end of each arm, photons are coupled into a single-mode fiber connected to a superconducting nanowire single-photon detector (SNSPDs-Single Quantum EOS, timing jitter $\le$ 25 ps), and a correlation electronics (IDQuantique ID800, temporal resolution 81 ps) that measures the difference in the arrival times of the two photons. The dark counts for signal and idler arms are approximately 300 Hz and 600 Hz, respectively.

## Results and discussion

**Figure 5:**
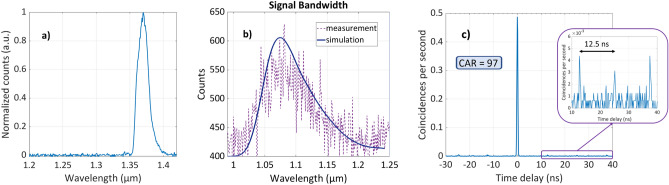
Correlation and spectrum measurement. (**a**) The experimentally measured spectrum of the pump pulse with the central wavelength of $\lambda _p$=1.37 $\upmu$m and (**b**) The experimentally measured signal spectrum for the x polarized pump and x polarized signal and idler. The solid line shows the simulation for the xxxx process that incorporates the measured pump pulse spectra. (**c**) Coincidence count rate as a function of time delay between signal and idler photons. The integration time for this measurement is 1600 s.

We perform classical spectroscopy of the signal photon using an InGaAs-detector-based spectrometer sensitive from 0.7 to 1.65 $\upmu$m, which cannot detect the idler photons at longer wavelengths, while it can detect the corresponding signal photons. To do this, we use a pump wavelength of $\lambda _p=1.37$ $\upmu$m polarized along x-direction to excite the process xxxx. Figure 5a shows the experimentally measured spectrum for this pump pulse, exhibiting the maximum at 1370 nm. In Fig. 5b, we show the signal spectrum for this pump pulse. Using the phase-matching condition in this fiber and the pump spectrum from the laser, we also calculate the expected signal photon spectrum, shown with a solid line in Fig. 5b. Details on the calculation can be found in references^52,63^. Note that the experimentally measured spectrum agrees well with the calculated signal photon spectrum from the phase-matching curves.

**Figure 6:**
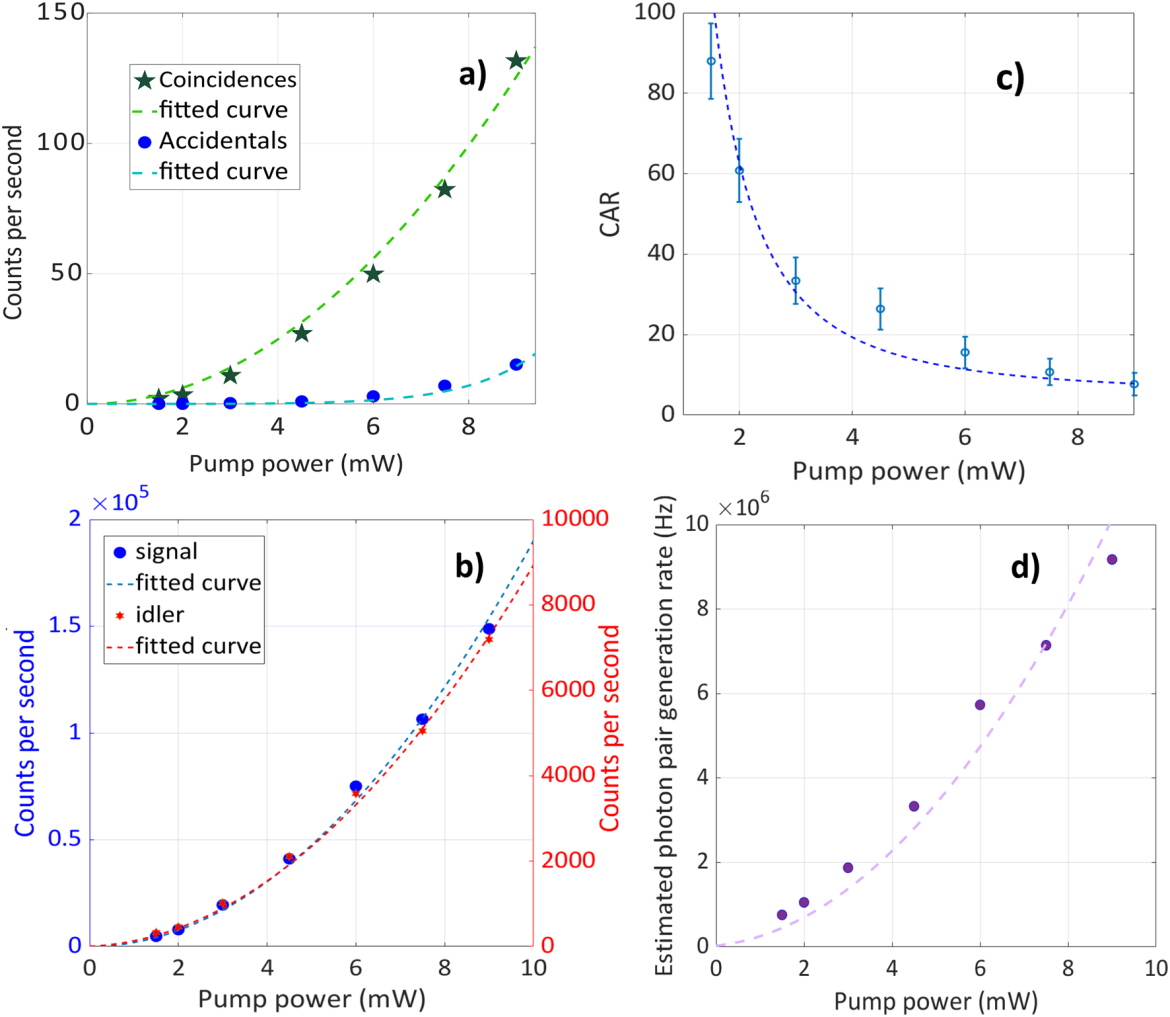
Power-dependent characteristics of photon pair generation, accidental and coincidence rates. (**a**) coincidence count rate and accidental count rate as a function of average pump power. The green dashed line represents a quadratic polynomial fit, while the blue dashed line represents a quartic polynomial fit. (**b**) single counts from the signal and idler channels as a function of average pump power. The dashed lines represent quadratic polynomial fit. (**c**) Coincidence-to-accidental ratio (CAR) as a function of the average pump power which decreases with the pump power as multi-pair generation dominates. (**d**) Estimated photon pair generation rate as a function of pump power. The dashed lines in (**c**) and (**d**) represent the fitted curves showcasing the evolution of CAR with 1/pump power^2^ and the photon pair generation rate with pump power^2^. The integration time is 300 s for all measurement points.

To demonstrate the generation of photon pairs from SFWM, correlation measurements were performed by recording the arrival times of photons at the single-photon detectors. Figure 5c represents the coincidence count rate per second as a function of the time delay between the signal and idler photons. The peak at the zero-delay time shows the strong temporal correlation between the photon detection events, verifying the generation of photon pairs. This dominant peak is mainly due to true coincidences, which come from correlated photon pairs. The smaller peaks are accidental coincidences coming from photons that simultaneously count on the two SPDs but do not come from the same pair. These peaks are separated by a multiple of 12.5 ns, which corresponds to the repetition rate of the laser (80 MHz). The coincidence count rate and the accidental count rate as functions of the average pump power are shown in Fig. 6a. The coincidence count rate follows a quadratic dependence when increasing the average pump power, which confirms a $\chi ^{(3)}$ process. To further investigate the properties of our fiber-based photon pair source, we measured the individual SFWM photon counts as a function of average pump power. Figure 6b shows the single count rate in the signal and idler channels as a function of average pump power on SPD1 and SPD2 with subtracted dark counts. As the SFWM process governs the pair generation, we observe a quadratic growth of the rate because the SFWM efficiency evolves with the square of the peak pump power.

A common measure for the quality of a photon-pair source is the so-called coincidence-to-accidental (CAR) ratio. This parameter is given as CAR $= (C_{m}-A)/A$, where $C_{m}$ is the total measured coincidence rate, and *A* is the accidental rate. The total measured coincidence rate per second ($C_{m}$) is determined from the total registered counts within the coincidence window and contains the accidental rate *A*. The number of counts in a secondary peak is equal to the number of accidental coincidences in the coincidence window. Therefore, we subtract *A* from $C_{m}$ in the numerator. The accidental rate is calculated from counts registered from the next coming pulse, corresponding to a 12.5 ns delay. Figure 6c shows the power dependency of the CAR. As the pump power increases, the CAR gradually decreases due to the increase in accidental counting rates by multiple photon-pair generation. The vertical error bar is estimated based on the Poissonian nature of the detection^70^. For an average pump power of 2 mW, we measured a CAR of around 97. A CAR value larger than 10 is generally accepted as useful for a fiber photon-pair source in quantum technology applications^11^, which makes our source a suitable one. Based on the measured single and coincidence counts, we estimated the total photon pair generation rate at the end of the fiber using $N_{pair} = N_s N_i / (C_m-A)$^71^, where $N_{s,i}$ is the single count rate in the signal/idler channel. In Fig. 6d, we plot the estimated photon-pair generation rate as a function of pump power.

Due to the numerous parameters involved in the interpretation of the obtained performance of a source, comparing the results and the performance of our fiber with other fiber structures is complex. However, here we achieve a CAR value larger than 90 and a generation efficiency of about $10^{-2}$ which is good in comparison to what is reported from other fiber-based sources^14–16,25–27,36,37,72,73^. We estimated the generation efficiency by dividing the pair generation rate by the pump pulse repetition rate. For such a high generation efficiency, the achieved CAR is very high and close to the expected fundamental limit from multiple pair generation, which scales with 1/pair generation rate^74^. It is important to note that with increasing the pump power, CAR is expected to decrease, while the generation efficiency is increasing. The collection efficiencies in the signal and idler channels are around 0.63% and 0.04%, respectively. These collection efficiencies have been estimated using $\eta _{s,i} = N_{s,i}/N_{pair}$, where $N_{pair}$ is the estimated photon pair generation rate, and $N_{s,i}$ are single counts for signal and idler.

We believe our CS_2_-filled microstructured optical fiber shows promising properties to contribute to developing fiber-based photon-pair sources.

## Conclusion

We experimentally demonstrated frequency non-degenerate photon-pair generation via spontaneous four-wave mixing in a novel CS_2_-filled microstructured optical fiber. We use CS_2_ as the core-filling liquid because it has high nonlinearity, narrow Raman lines, a broad transmission spectrum, and also allows for strong core-cladding index contrast. Moreover, using a microstructured liquid-core fiber in contrast to capillary-type liquid-core fiber results in a reduced fraction of power inside the silica. The phase-matching curve of this fiber offers a wide wavelength tunability based on the choice of the pump wavelength. For instance, as indicated by the tendency in Fig. 2c, tuning the pump wavelength to values below 1.3 micrometers enables the generation of signal photons in a spectral range where single-photon detection is typically achievable with high efficiency, using avalanche photodiodes. In this case, the corresponding idler photons will be in the short- and mid-wavelength infrared, which is not achievable in silica core fibers due to the silica loss in this range. This makes Li-COFs suitable sources for quantum spectroscopy schemes based on induced coherence^6,75^, where a photon detected at one wavelength reveals information about the transmission at another wavelength^1–4^. Thus, this fiber offers a potential application as a compact and efficient source for quantum spectroscopy and sensing. Moreover, with a demonstrated CAR larger than 90 and a generation efficiency of about $10^{-2}$, this work contributes to developing high-quality correlated photon pair sources for quantum technology applications, such as realizing quantum information processing protocols.

Additionally, because of the high nonlinearity of CS_2_ and strong transverse spatial mode confinement in the microstructured fiber, it becomes possible to engineer the dispersion relation for tailoring the spectral properties of the generated pair. By engineering the dispersion of the fiber, one can control the wavelength, group velocity, and bandwidths of two-photon states in liquid-filled microstructured fibers and generate a two-photon state with a specific spectral correlation. For instance, in refence^52^, the theoretical investigations have been conducted into the generation of photon pairs through SFWM in a liquid-filled microstructured suspended-core optical fiber. The study focuses on a design aimed at achieving a factorable state, enabling the heralding of a single-photon pure state without the need for spectral post-filtering. In conclusion, we believe this work contributes to further development of the advanced fiber-based sources for various quantum technology applications.

## Data Availability

Data underlying the results presented in this paper are not publicly available at this time but may be obtained from the corresponding author upon reasonable request.
